# Assessing and Quantifying the Surface Texture of Milk Powder Using Image Processing

**DOI:** 10.3390/foods11101519

**Published:** 2022-05-23

**Authors:** Haohan Ding, David I. Wilson, Wei Yu, Brent R. Young

**Affiliations:** 1 Science Center for Future Foods, Jiangnan University, Wuxi 214122, China; dinghaohan@jiangnan.edu.cn; 2 Department of Chemical & Materials Engineering, University of Auckland, Auckland 1010, New Zealand; w.yu@auckland.ac.nz (W.Y.); b.young@auckland.ac.nz (B.R.Y.); 3 Electrical and Electronic Engineering Department, Auckland University of Technology, Auckland 1010, New Zealand

**Keywords:** 3D image analysis, photogrammetry, surface smoothness, milk powder, surface normal analysis

## Abstract

Milk powders produced from similar spray dryers have different visual appearances, while the surface appearance of the powder is a key quality attribute because the smoothness of the milk powder also affects flowability and handling properties. Traditionally quantifying this nuanced visual metric was undertaken using sensory panelists, which is both subjective and time consuming. Therefore, it is advantageous to develop an on-line quick and robust appearance assessment tool. The aim of this work is to develop a classification model which can classify the milk powder samples into different surface smoothness groups. This work proposes a strategy for quantifying the relative roughness of commercial milk powder from 3D images. Photogrammetry equipment together with the software RealityCapture were used to build 3D models of milk powder samples, and a surface normal analysis which compares the area of the triangle formed by the 3 adjacent surface normals or compares the angle between the adjacent surface normals was used to quantify the surface smoothness of the milk powder samples. It was found that the area of the triangle of the smooth-surface milk powder cone is smaller than the area of the triangle of the rough-surface milk powder cone, and the angle between the adjacent surface normals of the rough-surface milk powder cone is larger than the angle between the adjacent surface normals of the smooth-surface milk powder cone, which proved that the proposed area metrics and angle metrics can be used as tools to quantify the smoothness of milk powder samples. Finally, the result of the support vector machine (SVM) classifier proved that image processing can be used as a preliminary tool for classifying milk powder into different surface texture groups.

## 1. Introduction

Product consistency and quality control are vital for the dairy industry [1], and visual consistency is important to customers. Thus, it is important to quantify visual texture grades, which include colour and roughness, for milk powder. However, maintaining visual consistency has proven to be difficult because different plants have differing process conditions which affect the powders’ appearance and properties [2,3,4]. Additionally, using trained sensory panellists to grade powders is both subjective and time-consuming. Therefore, an unbiased, efficient way to quantify surface appearance is desirable to rectify production problems and ensure a consistent looking product.

This study focuses solely on surface texture attributes. While various definitions of texture have been proposed to date [5], improved metrics and feature vectors are needed to quantify the roughness of the various powders. Massot-Campos et al. [6] point out that digital image analysis has the ability of quantifying surface environments and many researchers have employed related computer vision techniques to measure the surface roughness of engineering components [7,8,9,10], and various image processing techniques were used to obtain texture features from images in different applications [11,12,13]. For example, Garcıa et al. [11] used the Boolean model to extract the features from the binary images and analysis the grey level texture of these images, while Tsai and Hsieh [12] presented a method that can automatically inspect the defects in directionally textured surfaces by using the two-dimensional (2D) Fourier transform. Nevertheless, only two-dimensional image analysis was used in most of these studies [14,15,16,17,18]. One promising example is to exploit local variations in intensity such as using the grey level co-occurrence matrix (GLCM). This is a two-dimensional matrix with dimensions equal to the number of grey levels in an image [19]. This matrix depends on the number of occurrences of pixel pairs of given intensities at a given displacement. The GLCM has been widely used for texture analysis in different fields [20,21,22,23,24,25,26]. 

With improvements in 3D laser scanning and photogrammetry technology, there has been increasing interest in the use of three-dimensional images which have greater complexity and accuracy regarding material texture. Three-dimensional laser scanning is a method that can obtain three-dimensional data on the object surface contour by calculating the displacement on the camera sensor [27] and has been applied to texture analysis work on materials such as concrete and metal [28,29,30]. A cheaper alternative to laser scanning is to stitch together multiple photographs from different positions to reconstruct a 3D image. Thus, photogrammetry has become a feasible option in analysing surface texture [31]. It can be applied to maps or 3D reconstructions of the target of interest [32,33,34,35], although to date most photogrammetry has been applied to sediments and soil [36,37,38]. Merel and Farres [39] found that it is accurate enough to measure microrelief and surface evolution by using photogrammetry. In addition, Moret-Fernández et al. [40] proved that the volume and bulk density of small soil aggregates can be validly estimated by photogrammetry. Additionally, Rieke-Zapp and Nearing [38] found that using less automation is better for controlling image correlation, camera calibration and construction of digital elevation models, but Bertin et al. [41] argued that using automated software is better for the novice especially when the image quality is high. Manual systems also result in greater times required for processing the data [42,43,44].

To date, most studies on milk powder quality have focused on instrumental tests such as bulk density, particle size distribution, water activity and flowability [45,46]. For instance, Lee et al. [47] used equipment that can detect the electrical resistance difference between air and water to measure the wettability and dispersibility of milk powders, Nijdam and Langrish [48] used a graduated cylinder to calculate the bulk density of milk powders by calculating the change of volume after tapping, while Davenel et al. [49] characterized the rehydration of milk powders by using a kinetic pulse nuclear magnetic resonance (NMR) method. However, these instrumental tests do not indicate the sensory perception of these properties because the results detected by the instruments are different from the customers’ judgments of the milk powders [50,51]. In addition, various texture analysis methods have been employed. For example, Lille et al. [52] used sensory analysis to evaluate the appearance, texture and flavour of the snack products produced from whole milk powder and wholegrain rye flour. Gosselin et al. [53] used the GLCM method to analyse the texture of a blend of polymer powders. Jeon et al. [54] compared the texture and sensory properties of cream cheese and cholesterol-removed Cream cheese made from whole milk powder during 4 weeks storage while Ping et al. [55] investigated the influence of ice particle surface roughness on the retrieved cloud optical thickness and effective particle size. Additionally, scanning electron micrographs, powder electron diffraction and particle texture analysis (PTA) are also used to analyse the texture of different products [56,57,58]. Furthermore, Traill et al. [50] used a Rate-All-That-Applies (RATA) method to differentiate the visual appearance of different types of milk powders by using a trained sensory panel, and found that the degree of milk powder clumps is the key difference between milk powder samples. Therefore, Traill et al. [51] proposed a photographic standard which can be used by trained grading assessors to grade the clumpiness of multiple commercial dairy powders, and proved that this method can classify the dairy powders based on the level of visual clumpiness and can measure the dairy powders of more consistent appearance to supply to consumers’ preferences. However, grading the surface texture of milk powders by the sensory panel is subjective, and the three-dimensional models have more accurate information than the two-dimensional images. Furthermore, there are few relevant studies dealing with milk powders attempting to quantify surface roughness using 3D texture analysis, so the aim of this study is to explore the feasibility and reliability of using photogrammetry to classify the visual appearances of different milk powders. Photogrammetry equipment was combined with the software ‘*RealityCapture*’ to build 3D models of milk powder samples. Then surface normal analysis was used to process these 3D meshes. Finally, a support vector machine (SVM) classifier was developed to classify the milk powder samples into different surface smoothness groups, and the accuracy calculation was performed by using a confusion chart.

## 2. Methodology

### 2.1. Milk Powder Samples and Preparation

**Figure 1:**
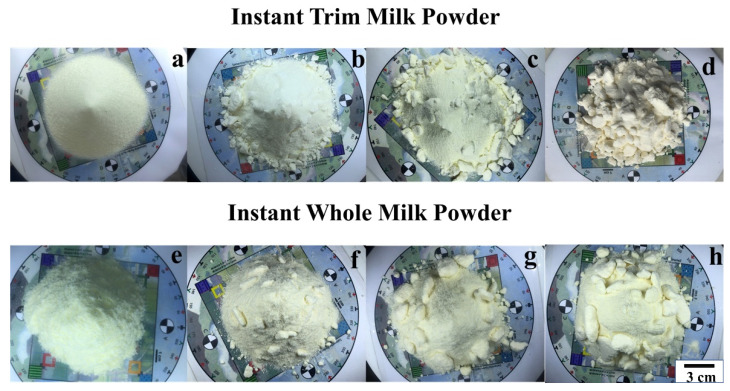
The plan (top) views of (**a**) Class 0 (**b**) Class 1 (**c**) Class 2 (**d**) Class 3 instant trim milk powder samples and (**e**) Class 0 (**f**) Class 1 (**g**) Class 2 (**h**) Class 3 instant whole milk powder samples.

The milk powders used in this study were bought from a local supermarket replicating the experience of a typical customer. The manufacturer of the milk powders is Fonterra Co-operative Group, and to investigate the effects of moisture on surface texture attributes of different kinds of milk powders, instant trim and instant whole milk powders were used in this work. The main differences between instant trim milk powder and instant whole milk powder are that the fat content of whole milk powder is higher than the fat content of trim milk powder and the whole milk powder is more heat sensitive than the trim milk powder [3]. Milk powder samples with different moisture levels may have different surface texture attributes, but the moisture level of milk powders purchased is approximately constant. Consequently, to synthesise powder samples with different moisture levels, (which approximates powder purchased at different times of the year, and at different geographical locations around the world), which in turn greatly affects the surface appearance, we sprayed varying amounts of water on the milk powder samples. Subsequent to the visual tests, these samples were oven-dried to establish the actual moisture [59,60]. In both the instant trim and the whole milk cases, four moisture levels were artificially fabricated, where it is noted that the samples with the first moisture level are the original, as-purchased, milk powder samples. The moisture levels (the mean of the three repeats with standard deviation) of all the milk powder samples are listed in Table 1, and the top or plan views of the different milk powder samples are shown in Figure 1. The plan (top) views of the milk powder samples in Figure 1 were compared with photo standards of different levels of clumpiness determined by Traill et al. [51]. The appearance of the milk powder samples with the first moisture level (samples a and e in Figure 1) are similar to the appearance of the level 0 dairy powders while the appearance of the milk powder samples with the fourth moisture level (samples d and h in Figure 1) are similar to the appearance of the level 14 (the extreme of the scale for milk powders) dairy powders. The milk powder samples with the first moisture level and the fourth moisture level were referred to as Class 0 and Class 3, respectively. Additionally, the appearance of the milk powder samples with the second moisture level (samples b and f in Figure 1) and the appearance of the milk powder samples with the third moisture level (samples c and g in Figure 1) should belong to the moderate clumping group (level 4–9) and the high clumping group (level 9–13), respectively. The milk powder samples with the second moisture level and the third moisture level were therefore referred to as Class 1 and Class 2, respectively. For each moisture level, three repetitions were taken. To make it easier for the photogrammetry software to distinguish specific reference points in what was otherwise a rather bland cone, Seurat’s *A Sunday Afternoon on the Island of La Grande Jatte* was used as a background image. This background image also includes four markers (A, B, C and D), 10° indicators around the circumference, and a 10 × 10 grid where each square is 1 cm × 1 cm. 

It is clear from Figure 1 that these milk powder samples are visually very different, and that the moisture level is strongly correlated to surface texture attributes, but it is necessary to find a repeatable, robust, mathematical way to quantify these differences. For repeatability, three milk powder cones were made for each moisture level. 

**Figure 2:**
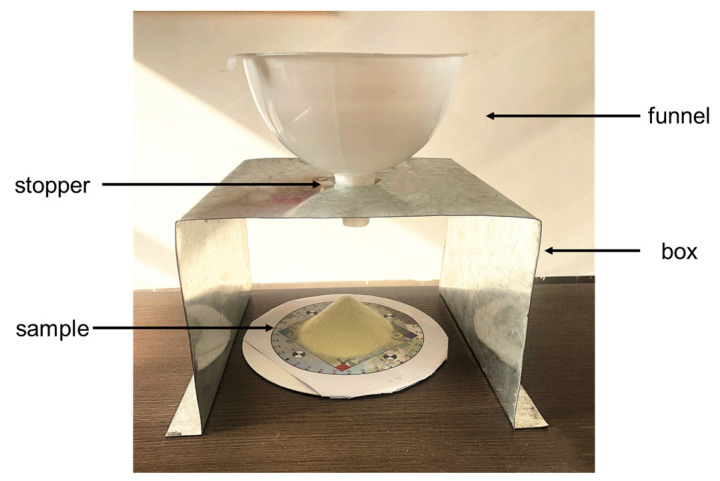
The milk powder delivery device shown ensures that the broad cone shape is reasonably consistent across all the samples.

The milk powder delivery device consists of a stopper, a funnel, and a box (shown in Figure 2), and to ensure the shape of each milk powder cone is similar, the device maintains the position of the funnel relative to the table. A stopper was placed in the funnel, and then, the funnel was filled with 80 g of milk powder. After that, the stopper was removed, and the funnel was tapped until the residue was minimal. For different milk powder samples, the shape of the top and bottom of the funnel used are the same. Since all the milk powder samples made are similarly shaped cones, it is assumed that the shape of the funnel used will not affect the sensitivity of the determinations. Additionally, the size of the bottom of the funnel may affect the size of the milk powder cone. Thus, an individual adjustment of the bottom of the funnel can be made if a different size of milk powder cone is required. Although the size of the milk powder cone will not affect its surface roughness, to make the milk powder samples more comparative, a similar size of milk powder cone is used.

### 2.2. Image Acquisition

**Figure 3:**
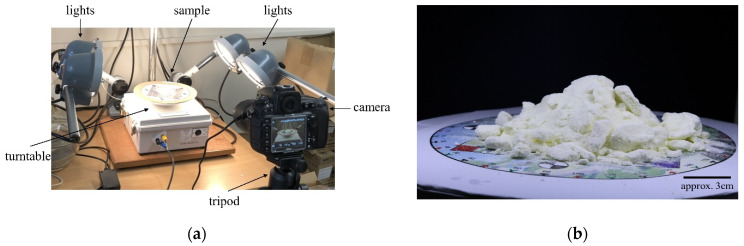
(**a**) The photogrammetry equipment setup with a milk powder sample; (**b**) An example photo of a Class 2 instant trim milk powder sample taken by the photogrammetry equipment from Figure 3a.

The photogrammetry equipment involved a camera, a tripod, a lighting system, and a turntable. The camera used was a Nikon D810 camera with a 60 mm lens, and it was fixed on the tripod which was 31 cm from the turntable. The height from the turntable to the center of the camera was about 28 cm. When taking photos, some settings of the camera are shooting modes: single, image quality: NEF (RAW), JPEG compression: optimal quality, white balance: AUTO, ISO sensitivity: 64, multiple exposure: OFF, interval timer shooting: OFF. The photogrammetry equipment is shown in Figure 3a. Four floodlights were used to obtain suitable lighting conditions for the image capture, and to eliminate the impact of shadows cast on the photos, the floodlights are placed in the four corners of the equipment. For photogrammetry, it is recommended to have at least 60% overlap between spatially successive images [61,62]. Consequently, images were taken every 11° resulting in 33 photos for each milk powder sample. To ensure that the reconstructed texture had no light or shadow information, the sample was rotated as opposed to the camera. Since the background of the photos may affect the 3D models of milk powder samples, a black cardboard was placed behind the milk powder sample so that the shape of the milk powder cones can be easily identified from the photos. Figure 3b shows an example photo of a Class 2 instant trim milk powder sample taken by this photogrammetry equipment.

### 2.3. Image Analysis

The computer used for all data processing was a Dell G3 3579 laptop which has an Intel^®^ core i7-8750H CPU @ 2.20 GHz with 16 GB installed RAM. The graphics card of the laptop is NVIDIA GeForce GTX 1060 with Max-Q Design.

**Figure 4:**
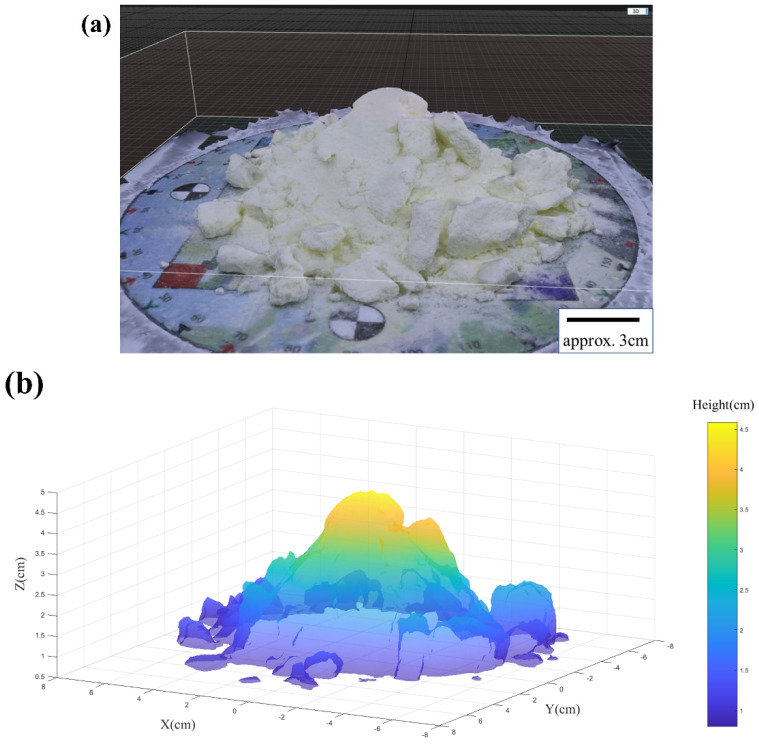

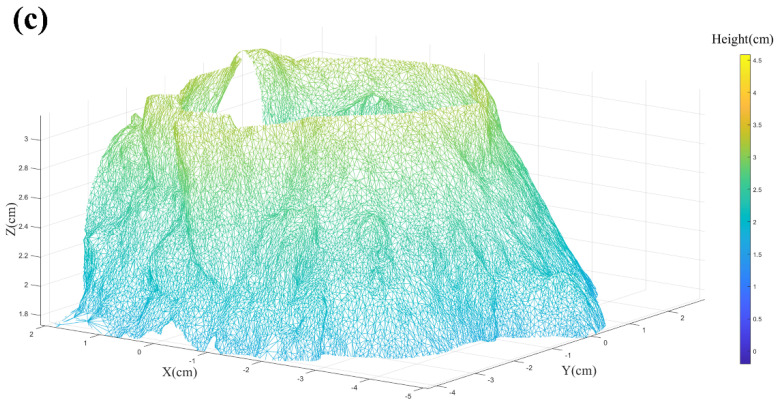
(**a**) An example of the reconstructed 3D mesh model of a Class 2 instant trim milk powder sample; (**b**) The transparent triangulation 3D mesh model of a Class 2 instant trim milk powder sample; (**c**) An enlarged portion of the same cone from (**b**), but this time showing the individual triangulated mesh.

The first step in image analysis involved building the 3D model as a triangular mesh using the software *RealityCapture* [63,64] from 33 photographs. After acquiring photos by using the photogrammetry equipment, the 33 photos were inputted into the software RealityCapture. Before creating a complete 3D reconstruction with texture using all inputs, the application settings for options were checked. The generated fine resolution mesh of around 7,000,000 triangles was downsampled by a factor of about 10 due to the computational constraints of the laptop as shown in Figure 4a. Subsequently, the triangular mesh model was exported to *Matlab* as shown in Figure 4b. Figure 4c gives a sense of the triangular mesh resolution.

### 2.4. Surface Normal Analysis

Given the high-resolution triangulation of the surface, one strategy to quantify roughness is to measure the difference between the surface normals on adjacent triangles. Whether the local surface is concave or convex, the normals are inward or outward, the surface normals will point in similar directions when the local surface is perfectly flat. While the surface normals will point in different directions when the local surface is rough.

#### 2.4.1. Comparing the Area of Triangle Formed by the 3 Adjacent Surface Normals

**Figure 5:**
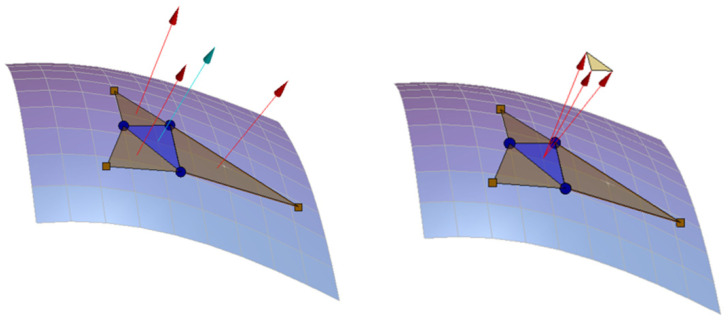
The area of the triangle formed by the 3 adjacent surface normals to the central triangle of interest (blue). If the entire surface is smooth, then the median of all the areas will be small.

The 3 adjacent surface normals of any given triangle are normalised, and then transferred to the origin in order to quantify the local smoothness (shown in Figure 5). The 3 surface normals will point in the same directions and the area of the triangle formed by the 3 normals will be zero when the local surface is flat. Conversely, as shown in Figure 5, if the surface is not flat, the area given by the three vectors will be non-zero. The median area of this subtended triangle for all interior mesh triangles gives a scalar value related to the surface texture attributes.

#### 2.4.2. Comparing the Angle between the Adjacent Surface Normals

**Figure 6:**
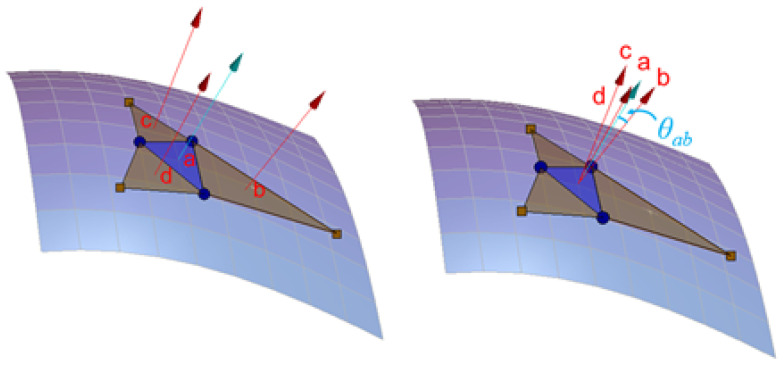
The angle between the surface normal of the base triangle and the adjacent surface normals.

An alternative method of characterizing the surface smoothness is to compute the angle between the surface normal of the base triangle, (i.e., the blue triangle in Figure 6), and the adjacent surface normal (as shown in Figure 6). The angle between vector **a** and **b** measured from the tail of **a** to the tail of **b** is given by the dot product,

(1) $$\theta_{ab}=\cos^{-1}(\frac{a\cdot b}{\left|a||b\right|})$$

Considering the computational load of the laptop (each sample has 500,000 triangles), this is simplified in this study given that all the surface normals calculated have a magnitude of 1. The angles between the base normal and the adjacent surfaces normals were calculated (*θ_ab_*, *θ_ac_* and *θ_ad_*), and the median value of these 3 angles was used. Furthermore, the data of the triangles on the boundary or the data whose value is zero were deleted.

### 2.5. Data Analysis

#### 2.5.1. Principal Component Analysis (PCA)

Principal component analysis (PCA) is a useful technique to reduce the dimension of a data set that consists of many interrelated data points and to keep most of the data information [65]. It has been used in many scientific fields [66]. In this research, given a cumulative distribution profile, (say a discretized empirical cumulative distribution function or ECDF), it would be useful to automatically classify the powder into different surface smoothness groups. The inputs to the classifier are values taken from the ECDF profiles for the area and angle metrics. The values used are the 9 equally spaced percentiles from 10% to 90% for the area, *A*_0.1_ to *A*_0.9_, and angle, *θ*_0.1_ to *θ*_0.9_ giving 18 variables in total. With so many variables, this classification problem is in danger of becoming over-parameterized, and hence fragile, so a PCA decomposition was used to reduce the dimension of the problem and optionally select a reduced set of variables for the classifier.

#### 2.5.2. Support Vector Machine (SVM)

The support vector machine method, (SVM), is a kernel-based technique that was developed for the binary classification problem [67], and pattern recognition problems [68]. In this study, after trying a variety of classifiers, the nonlinear SVM gave the best result. Consequently, a 3rd order polynomial nonlinear SVM was developed to classify the surface smoothness of milk powder samples. In this study, cross-validation (CV) [69] was used to avoid overfitting the model. Six data sets (75% of the data) were used for training the model, while two data sets (25% of the data) were used for validation. The model that outperformed the others was selected to classify the surface smoothness of milk powders. The definition and calculation of the performance parameters of the confusion chart, such as overall accuracy, sensitivity and specificity, are described in [70,71].

## 3. Results and Discussion

### 3.1. The Comparison of Areas of Triangles

**Figure 7:**
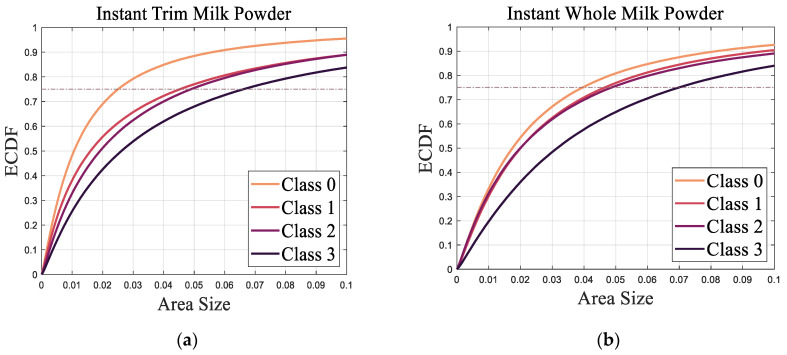
(**a**) ECDF of areas of triangles for instant trim milk powders; (**b**) ECDF of areas of triangles for instant whole milk powders.

The ECDF of all the areas of triangles for the Classes 0–3 of the instant trim and whole milk powder samples are shown in Figure 7a,b, respectively. Since for each moisture level, there are three milk powder samples, the average ECDF values of the areas of triangles is presented in Figure 7, and the 75 percentile values were chosen to extract a scalar area metric from the (vector) ECDF.

**Figure 8:**
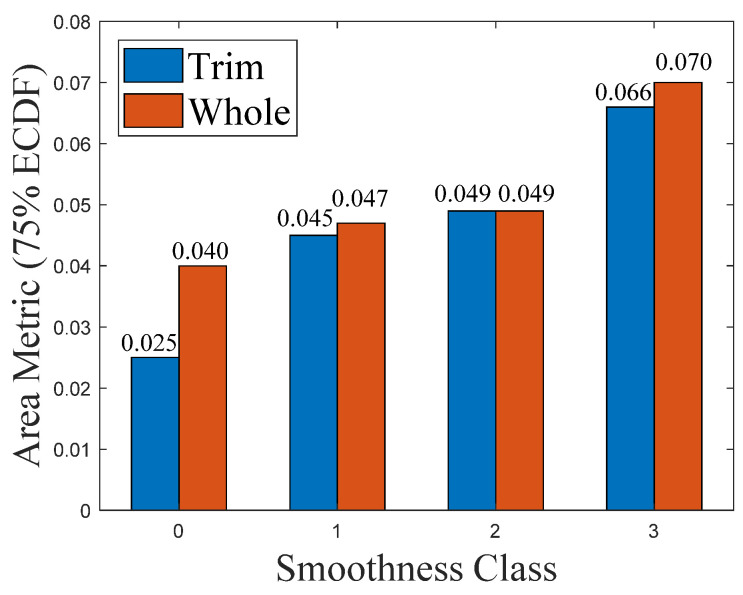
The area metric versus the smoothness class of instant trim and whole milk powder.

Figure 8 presents the relationship between the area metric and the smoothness class of instant trim and whole milk powder samples.

What is clear is that Class 3 samples have a higher proportion of larger areas than other classes and the scalar metrics (75 percentile ECDF value) of Class 0 samples are smaller than the scalar metrics of other classes, which is as expected. In addition, the 75 percentile ECDF value of the Class 0 instant whole milk powder is much higher than the Class 0 instant trim milk powder. In practice, these results can be proved by Figure 1, which shows that Class 3 samples are more lumpy than other classes, and the surfaces of the Class 0 samples are smoother than the surfaces of other classes. Additionally, the surface of Class 0 instant trim milk powder sample is obviously smoother than the surface of Class 0 instant whole milk powder sample, and this may be because the particles of instant whole milk powder have greater agglomeration and become more irregular in shape [2,46].

### 3.2. The Comparison of Angles between the Adjacent Surface Normals

**Figure 9:**
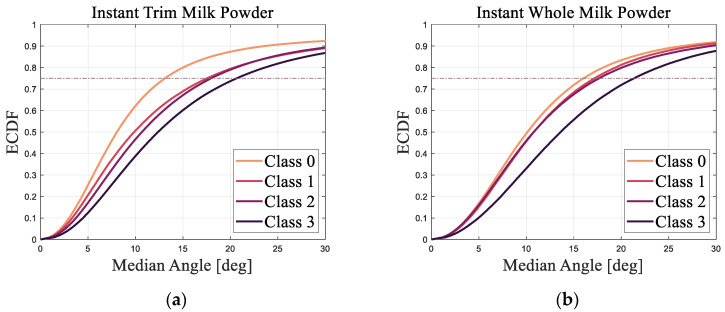
(**a**) The empirical cumulative distribution function of all the angles between the adjacent surface normals for Classes 0–3 samples of instant trim milk powder; (**b**) The empirical cumulative distribution function of all the angles between the adjacent surface normals for Classes 0–3 samples of instant whole milk powder.

In a similar manner to the approach taken above, the ECDF of all the *angles* between the surface normal of the base triangle and the surface normal of the adjacent triangle for Classes 0–3 instant trim and whole milk powder samples are illustrated in Figure 9a,b, respectively. The ECDF values in Figure 9 are also the average ECDF values of the angles of three milk powder samples for different moisture levels.

**Figure 10:**
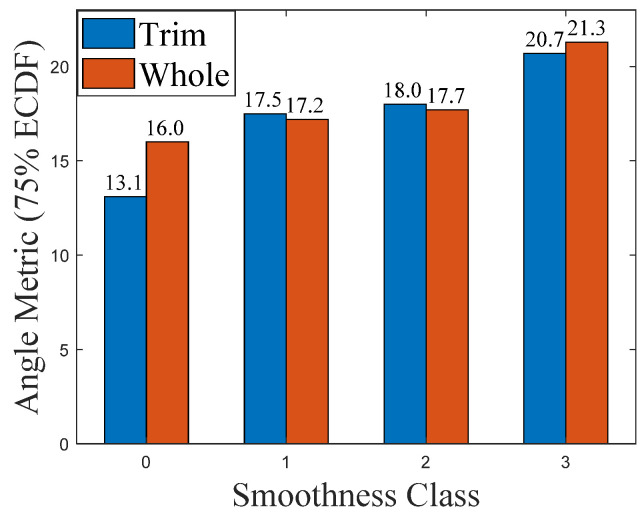
The angle metric versus the smoothness class of instant trim and whole milk powder.

The 75th percentile value of the ECDF was chosen as the scalar angle metric and Figure 10 shows the angle metric versus the smoothness class of instant trim and whole milk powder.

From Figure 9, it is notable that Class 0 samples have a higher proportion of smaller angles than other classes. Additionally, the ECDF curves of Class 3 milk powder samples are obviously lower than the ECDF curves of Classes 0–2 milk powder samples, while the ECDF values of Class 1 milk powder samples are slightly higher than the ECDF values of Class 2 milk powder samples. These results show that the surface of Class 3 milk powder samples is rougher than the surface of Classes 0–2 milk powder samples and the surface of Class 0 milk powder samples is the smoothest. Furthermore, the angle metric of Class 0 instant whole milk powder sample is larger than the angle metric of the Class 0 instant trim milk powder sample, indicating that the surface of the instant whole milk powder sample is rougher than the surface of the instant trim milk powder sample. Finally, the results of the area metrics and angle metrics presented in Figure 8 and Figure 10 show that using photogrammetry and surface normal analysis to distinguish milk powder samples with different surface smoothness is feasible.

From the results, the overall trends of the areas of triangles for the Classes 0–3 of the instant trim milk powder samples are similar to the trends of the areas of triangles for the Classes 0–3 of the instant whole milk powder samples, while the overall trends of the angles between the adjacent surface normals for Classes 0–3 instant trim milk powder samples are also similar to the trends of the angles between the adjacent surface normals for Classes 0–3 instant whole milk powder samples. This indicates that the selected metrics can classify the surface smoothness of different types of milk powders. Furthermore, the overall trends of the areas of triangles for milk powder samples are similar to the overall trends of the angles between the adjacent surface normals of milk powder samples, which proves that the angle metric is a good substitute for the area metric.

**Figure 11:**
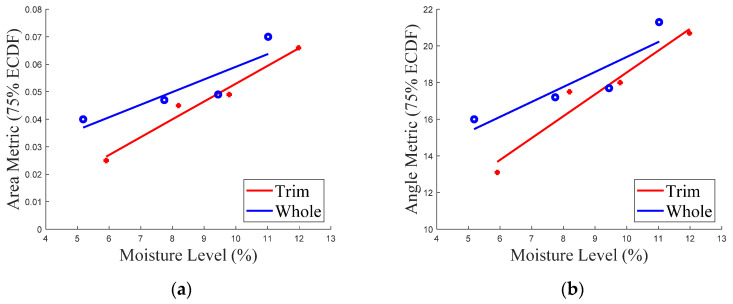
(**a**) The area metric versus the moisture level of instant trim and whole milk powder; (**b**) The angle metric versus the moisture level of instant trim and whole milk powder.

While it was not the original intention of this study, it is interesting to note that the moisture level is strongly correlated to surface texture attributes. This result is of course not unexpected and is precisely the rationale for how differing roughness samples were synthesized from the same powder by adjusting the moisture. The trends of area metric versus the change of moisture level and angle metric versus the change of moisture level for instant trim and whole milk powder are shown in Figure 11a,b, respectively. The area metric and the angle metric of instant trim and whole milk powder exhibit a positive correlation (*R*^2^ values of the curves in Figure 11a are 0.97 and 0.79, while *R*^2^ values of the curves in Figure 11b are 0.94 and 0.80, respectively), which means that increasing the moisture level, increases the surface roughness. This result demonstrates that the area and angle metrics can be used as the suitable tools to quantify the smoothness of milk powder samples [72,73].

### 3.3. Classification of the Surface Smoothness

**Figure 12:**
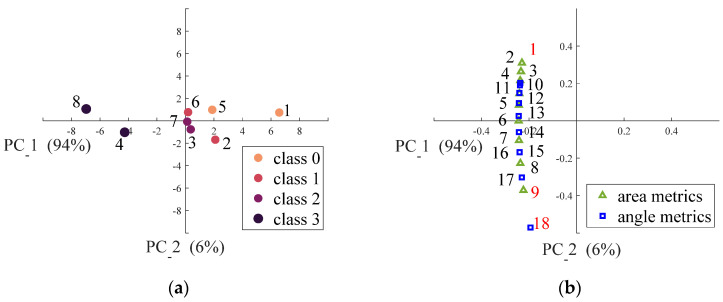
(**a**) PCA scores plot of the first two principal components distinguishing the eight milk powder samples from their surface smoothness; (**b**) PCA loadings plot of the first two principal components distinguishing the relations between angle metrics and area metrics.

The PCA scores and loadings plots are shown in Figure 12a,b, respectively. The points labelled 1 to 4 in Figure 12a represent, separately, Class 0, Class 1, Class 2, and Class 3 instant trim milk powder samples, while the points labeled 5 to 8 represent, respectively, Class 0, Class 1, Class 2, and Class 3 instant whole milk powder samples. From Figure 12a, PC1 and PC2 explain 94% and 6% of the total variance, respectively. Class 0 instant trim milk powder sample (sample 1) and Class 3 instant trim and whole milk powder samples (sample 4 and sample 8) were well separated with their surface smoothness along PC1, because the surface of Class 0 instant trim milk powder is the smoothest while the surface of Class 3 instant trim and whole milk powder samples is the roughest. However, Class 0 instant whole milk powder sample (sample 5), Class 1 instant trim and whole milk powder samples (sample 2 and sample 6) and Class 2 instant trim and whole milk powder samples (sample 3 and sample 7) were close to each other, which indicates that the surface smoothness of these milk powder samples are similar. Additionally, the points labelled 1 to 18 in Figure 12b, respectively, represent the ECDF values of the area metrics and the ECDF values of the angle metrics (*A*_0.1_ to *A*_0.9_ and *θ*_0.1_ to *θ*_0.9_). From Figure 12b, points 1 (*A*_0.1_), 9 (*A*_0.9_) and 18 (*θ*_0.9_) have relatively high loadings. Therefore, these three variables were selected to develop the classification model to classify the smoothness of milk powder samples.

**Figure 13:**
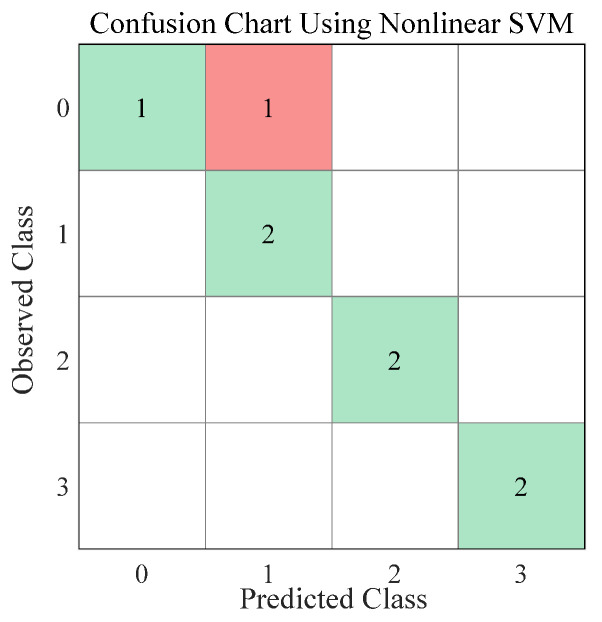
The confusion chart for the nonlinear SVM classifier.

The prediction results of the SVM are presented in Figure 13. The true positives (TP) and true negatives (TN) from the predictions are represented as green cells, while the red parts represent the numbers of the false negative (FN) and false positive (FP) predictions. From these results, the overall accuracy of the nonlinear SVM is approximately 88%, and the sensitivity and specificity of the nonlinear SVM classifier is presented in Table 2. Additionally, from the results of the classifier, only one Class 0 milk powder sample (sample 5) was wrongly predicted as a Class 1 sample which may be because the surface of the Class 0 instant whole milk powder sample is rougher than the surface of the Class 0 instant trim milk powder sample, and the surface smoothness of Class 0 instant whole milk powder sample is similar to the surface smoothness of Class 1 milk powder samples. Of course, the user can adjust the threshold between different surface smoothness levels if desired. Finally, this good SVM result proved that using this image processing method as a preliminary tool for classifying milk powder into different surface texture groups is feasible.

## 4. Conclusions

By constructing 3D models of milk powder samples and analyzing the surface normals of 3D models, the current research aims to explore the feasibility of using photogrammetry for visual appearances classification of different milk powders. The proposed methodology is objective and can improve plant efficiency as an alternative to traditional visual attributes measurement. However, the current study only has four appearance grades of milk powder. To make the classifier more accurate and robust, it is recommended to have more appearance grades of milk powder in future research.

The application of image processing for surface appearance grading was investigated using two types of commercially available off-the-shelf milk powders. Photogrammetry equipment combined with the software *RealityCapture* was used to rapidly and economically develop the 3D mesh models which then could be readily post-processed in *Matlab* to extract various quality metrics.

The metrics that are chosen in this study relied on the fact that the area of the triangle of the rough-surface milk powder cone is larger than the area of the triangle of the smooth-surface milk powder cone, and the angle between the adjacent surface normals of the smooth-surface milk powder cone is smaller than the angle between the adjacent surface normals of the rough-surface milk powder cone. Therefore, the surface normal analysis was effective for quantifying the surface appearance of milk powders, and this method may offer a more robust way of classifying milk powders that have different surface appearances, especially the lumpiness of the cone. Lastly, the good result from the SVM proved that the image processing methods proposed here are feasible alternative methods for classifying the visual appearance of the powders.

## Figures and Tables

**Table 1:** The moisture levels (% by weight) of each milk powder sample.

Moisture	Instant Trim Milk Powder	Instant Whole Milk Powder
Class 0 (original)	5.91 ± 0.27%	5.18 ± 0.23%
Class 1	8.19 ± 0.45%	7.74 ± 0.51%
Class 2	9.79 ± 0.66%	9.44 ± 0.64%
Class 3	11.98 ± 0.96%	11.02 ± 0.87%

**Table 2:** The sensitivity and specificity of the nonlinear SVM classifier.

Class	Sensitivity	Specificity
Class 0	50 %	100 %
Class 1	100 %	83 %
Class 2	100 %	100 %
Class 3	100 %	100 %

## Data Availability

Not applicable.
